# *fac*-Tris(pyridine-2-carboxyl­ato-κ^2^ *N*,*O*)cobalt(III)

**DOI:** 10.1107/S1600536811043303

**Published:** 2011-10-29

**Authors:** Irina A. Golenia, Alexander N. Boyko, Natalia V. Kotova, Matti Haukka, Valentina A. Kalibabchuk

**Affiliations:** a Department of Chemistry, Kiev National Taras Shevchenko University, Volodymyrska Street 64, 01601 Kiev, Ukraine; b Department of Chemistry, University of Joensuu, PO Box 111, FI-80101 Joensuu, Finland; c Department of General Chemistry, O. O. Bohomolets National Medical University, Shevchenko Boulevard 13, 01601 Kiev, Ukraine

## Abstract

In the title compound, [Co(C_6_H_4_NO_2_)_3_], the Co^III^ ion lies on a threefold rotation axis and is in a distorted octa­hedral environment defined by three N and three O donor atoms from three *fac*-disposed pyridine-2-carboxyl­ate ligands. The ligands are coordinated in a chelate fashion, forming three five-membered rings. In the crystal, translationally related complex molecules are organized into columns along [001] *via* C—H⋯O hydrogen bonds.

## Related literature

For the use of hydroxamate ligands in the synthesis of polynuclear compounds, see: Dobosz *et al.* (1999 ▶); Fritsky *et al.* (1998 ▶); Sachse *et al.* (2008 ▶). For hydrolytic destruction of hydroxamate ligands upon complex formation, see: Świątek-Kozłowska *et al.* (2000 ▶). For related structures, see: Fritsky *et al.* (2001 ▶); Fu & Wang (2005 ▶); Kovbasyuk *et al.* (2004 ▶); Krämer & Fritsky (2000 ▶); Mokhir *et al.* (2002 ▶); Moroz *et al.* (2010 ▶); Pelizzi & Pelizzi (1981 ▶); Sliva *et al.* (1997 ▶); Wörl, Fritsky *et al.* (2005 ▶); Wörl, Pritzkow *et al.* (2005 ▶). For the synthesis of pyridine-2-hydroxamic acid, see: Hynes (1970 ▶).
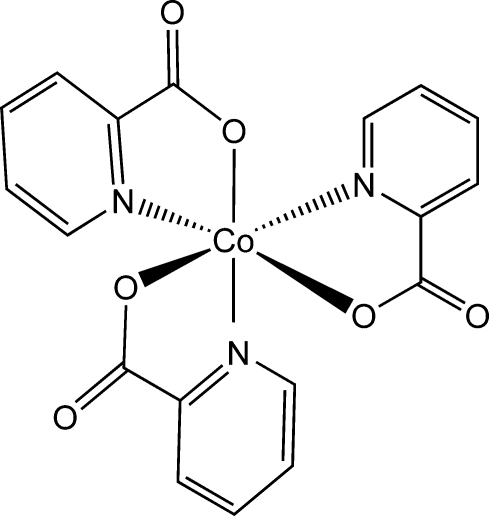


## Experimental

### 

#### Crystal data

[Co(C_6_H_4_NO_2_)_3_]
*M* *_r_* = 425.24
Hexagonal, 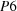

*a* = 12.8617 (12) Å
*c* = 6.2122 (9) Å
*V* = 890.0 (2) Å^3^
*Z* = 2
Mo *K*α radiation
μ = 1.01 mm^−1^
*T* = 120 K
0.23 × 0.08 × 0.03 mm

#### Data collection

Nonius KappaCCD diffractometer
Absorption correction: multi-scan (*DENZO*/*SCALEPACK*; Otwinowski & Minor, 1997 ▶) *T*_min_ = 0.800, *T*_max_ = 0.970
5635 measured reflections
978 independent reflections
893 reflections with *I* > 2σ(*I*)
*R*_int_ = 0.043

#### Refinement

*R*[*F* ^2^ > 2σ(*F* ^2^)] = 0.068
*wR*(*F* ^2^) = 0.197
*S* = 1.16
978 reflections
86 parameters
1 restraint
H-atom parameters constrained
Δρ_max_ = 1.05 e Å^−3^
Δρ_min_ = −0.59 e Å^−3^
Absolute structure: Flack (1983 ▶), 400 Friedel pairs
Flack parameter: −0.02 (7)

### 

Data collection: *COLLECT* (Nonius, 1998 ▶); cell refinement: *DENZO*/*SCALEPACK* (Otwinowski & Minor, 1997 ▶); data reduction: *DENZO*/*SCALEPACK*; program(s) used to solve structure: *SIR2004* (Burla *et al.*, 2005 ▶); program(s) used to refine structure: *SHELXL97* (Sheldrick, 2008 ▶); molecular graphics: *DIAMOND* (Brandenburg, 1999 ▶); software used to prepare material for publication: *SHELXL97*.

## Supplementary Material

Crystal structure: contains datablock(s) I, global. DOI: 10.1107/S1600536811043303/hy2479sup1.cif

Structure factors: contains datablock(s) I. DOI: 10.1107/S1600536811043303/hy2479Isup2.hkl

Additional supplementary materials:  crystallographic information; 3D view; checkCIF report

## Figures and Tables

**Table 1:** Hydrogen-bond geometry (Å, °)

*D*—H⋯*A*	*D*—H	H⋯*A*	*D*⋯*A*	*D*—H⋯*A*
C3—H3⋯O2^i^	0.95	2.60	3.212 (14)	123

Symmetry code: (i) 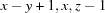.

**Fig. 1.**
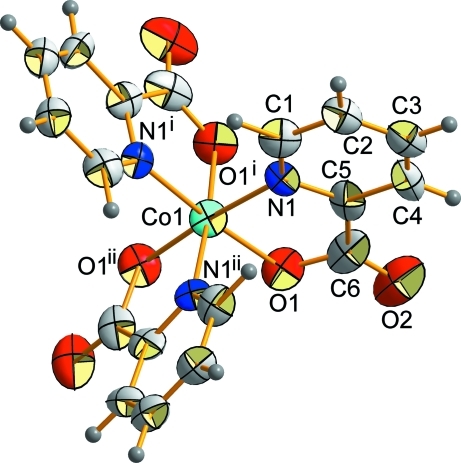
The molecular structure of the title compound. Displacement ellipsoids are shown at the 50% probability level. [Symmetry codes: (i) -x+y, 1-x, z; (ii) 1-y, 1+x-y, z.]

**Fig. 2.**
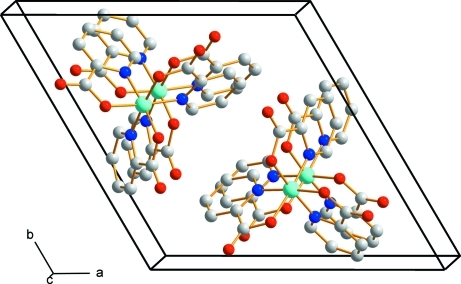
A packing diagram of the title compound. H atoms have been omitted for clarity.

## supplementary crystallographic information

### Comment

Hydroxamic groups and their derivatives are often used in syntheses of polynuclear metal complexes (Dobosz *et al.*, 1999; Fritsky *et al.,* 1998; Sachse *et al.*, 2008). However, functionalized hydroxamate ligands having additional donor functions often undergo hydrolytic destruction when complex formation with 3d-metal ions takes place (Dobosz *et al.*, 1999; Świątek-Kozłowska *et al.*, 2000). As a result, a carboxylic group is formed. The title compound was obtained as a result of hydrolytic decomposition of pyridine-2-hydroxamic acid during the reaction of complex formation with cobalt(II) perchlorate.

In the title compound, the Co^III^ ion lies on a threefold rotation axis and is in a distorted octahedral environment of three N and three O donor atoms from three pyridine-2-carboxylate ligands (Fig. 1). Unlike in the case of earlier reported tris(pyridine-2-carboxylato)cobalt(III) monohydrate, in which the realization of *mer*-isomer is observed, in the title complex three pyridine-2-carboxylate ligands are disposed in a *fac*-fashion (Fu & Wang, 2005; Pelizzi & Pelizzi, 1981). The ligands are coordinated in a chelate mode, forming three five-membered rings. The Co—N and Co—O bond lengths are consistent with the values typically quoted for the octahedral cobalt(III) complexes with N,O-mixed donor ligands (Mokhir *et al.*, 2002; Sliva *et al.*, 1997). The C—O bond lengths in the deprotonated carboxylate groups of the ligands differ significantly [1.232 (14) and 1.339 (15) Å], which is typical for monodentately coordinated carboxylates (Wörl, Fritsky *et al.*, 2005; Wörl, Pritzkow *et al.*, 2005). The C—C and C—N bond lengths in the pyridine rings are normal for 2-substituted pyridine derivatives (Fritsky *et al.,* 2001; Kovbasyuk *et al.*, 2004; Krämer & Fritsky, 2000; Moroz *et al.*, 2010).

In the crystal, the translational complex molecules are organized into columns along the *c* axis (Fig. 2). The neighboring molecules are united by C—H···O hydrogen bonds (Table 1).

### Experimental

Cobalt(II) perchlorate hexahydrate (0.0365 g, 0.1 mmol) was dissolved in water (3 ml) and mixed with a solution of pyridine-2-hydroxamic acid (0.0414 g, 0.3 mmol) (Hynes, 1970) in methanol (3 ml). The resulting clear red solution was set aside for crystallization by slow diffusion of diethyl ether into the formed solution. The pink crystals formed in 5–7 days were filtered off, washed with diethyl ether and air-dried (yield: 83%).

### Refinement

The final structure refinement was performed by using a twin law (-1 -1 0 0 1 0 0 0 -1) with the final BASF parameter refining to 0.80178. H atoms were positioned geometrically and refined as riding atoms, with C—H = 0.95 Å and with *U*_iso_(H) = 1.2*U*_eq_(C).

### Crystal data

**Table:**

[Co(C_6_H_4_NO_2_)_3_]	*D*_x_ = 1.587 Mg m^−^^3^
*M**_r_* = 425.24	Mo *K*α radiation, λ = 0.71073 Å
Hexagonal, *P*6	Cell parameters from 713 reflections
Hall symbol: P 6	θ = 3.2–24.5°
*a* = 12.8617 (12) Å	µ = 1.01 mm^−^^1^
*c* = 6.2122 (9) Å	*T* = 120 K
*V* = 890.0 (2) Å^3^	Block, pink
*Z* = 2	0.23 × 0.08 × 0.03 mm
*F*(000) = 432	

### Data collection

**Table:**

Nonius KappaCCD diffractometer	978 independent reflections
Radiation source: fine-focus sealed tube	893 reflections with *I* > 2σ(*I*)
horizontally mounted graphite crystal	*R*_int_ = 0.043
Detector resolution: 9 pixels mm^-1^	θ_max_ = 25.0°, θ_min_ = 3.2°
φ and ω scans with κ offset	*h* = −15→15
Absorption correction: multi-scan (*DENZO*/*SCALEPACK*; Otwinowski & Minor, 1997)	*k* = −15→15
*T*_min_ = 0.800, *T*_max_ = 0.970	*l* = −7→7
5635 measured reflections	

### Refinement

**Table:**

Refinement on *F*^2^	Secondary atom site location: difference Fourier map
Least-squares matrix: full	Hydrogen site location: inferred from neighbouring sites
*R*[*F*^2^ > 2σ(*F*^2^)] = 0.068	H-atom parameters constrained
*wR*(*F*^2^) = 0.197	*w* = 1/[σ^2^(*F*_o_^2^) + (0.1316*P*)^2^ + 0.9442*P*] where *P* = (*F*_o_^2^ + 2*F*_c_^2^)/3
*S* = 1.16	(Δ/σ)_max_ < 0.001
978 reflections	Δρ_max_ = 1.05 e Å^−^^3^
86 parameters	Δρ_min_ = −0.59 e Å^−^^3^
1 restraint	Absolute structure: Flack (1983), 400 Friedel pairs
Primary atom site location: structure-invariant direct methods	Flack parameter: −0.02 (7)

### Special details

**Table:**

Experimental. The final structural refinement was performed by using the twin law -1 -1 0 0 1 0 0 0 -1 with the final BASF parameter refining to 0.80178.

### Fractional atomic coordinates and isotropic or equivalent isotropic displacement parameters (Å^2^)

**Table:**

	*x*	*y*	*z*	*U*_iso_*/*U*_eq_	
Co1	0.3333	0.6667	0.3408 (2)	0.0379 (5)	
O1	0.4550 (7)	0.7860 (7)	0.5147 (11)	0.0514 (18)	
O2	0.6490 (9)	0.8907 (8)	0.5627 (16)	0.083 (3)	
N1	0.4619 (6)	0.6785 (6)	0.1705 (12)	0.0332 (15)	
C1	0.4622 (10)	0.6303 (9)	−0.0009 (17)	0.051 (2)	
H1	0.3875	0.5756	−0.0656	0.061*	
C2	0.5687 (9)	0.6533 (9)	−0.1037 (17)	0.050 (2)	
H2	0.5659	0.6145	−0.2355	0.061*	
C3	0.6747 (10)	0.7308 (9)	−0.0138 (18)	0.052 (2)	
H3	0.7483	0.7480	−0.0795	0.062*	
C4	0.6729 (9)	0.7796 (9)	0.159 (2)	0.052 (3)	
H4	0.7469	0.8358	0.2241	0.062*	
C5	0.5690 (8)	0.7548 (9)	0.2577 (18)	0.048 (2)	
C6	0.5660 (11)	0.8159 (9)	0.4512 (18)	0.057 (3)	

### Atomic displacement parameters (Å^2^)

**Table:**

	*U*^11^	*U*^22^	*U*^33^	*U*^12^	*U*^13^	*U*^23^
Co1	0.0513 (7)	0.0513 (7)	0.0111 (8)	0.0256 (3)	0.000	0.000
O1	0.067 (4)	0.066 (4)	0.018 (4)	0.031 (4)	−0.007 (3)	−0.008 (3)
O2	0.105 (7)	0.067 (5)	0.054 (6)	0.027 (4)	−0.036 (5)	0.004 (4)
N1	0.045 (4)	0.047 (4)	0.014 (3)	0.028 (3)	−0.001 (3)	0.007 (3)
C1	0.065 (5)	0.056 (5)	0.034 (5)	0.031 (4)	0.006 (4)	0.008 (4)
C2	0.061 (5)	0.058 (5)	0.041 (6)	0.036 (5)	0.010 (4)	0.006 (4)
C3	0.060 (6)	0.059 (6)	0.046 (6)	0.038 (5)	0.014 (5)	0.016 (5)
C4	0.042 (5)	0.067 (6)	0.055 (7)	0.033 (4)	0.012 (5)	0.023 (6)
C5	0.048 (5)	0.057 (5)	0.042 (6)	0.029 (4)	−0.005 (4)	0.021 (5)
C6	0.071 (7)	0.052 (6)	0.036 (6)	0.021 (5)	−0.027 (6)	0.010 (5)

### Geometric parameters (Å, °)

**Table:**

Co1—O1^i^	1.889 (7)	C1—C2	1.402 (14)
Co1—O1	1.889 (7)	C1—H1	0.9500
Co1—O1^ii^	1.889 (7)	C2—C3	1.344 (16)
Co1—N1^i^	1.904 (7)	C2—H2	0.9500
Co1—N1^ii^	1.904 (7)	C3—C4	1.251 (15)
Co1—N1	1.904 (7)	C3—H3	0.9500
O1—C6	1.339 (15)	C4—C5	1.354 (14)
O2—C6	1.232 (14)	C4—H4	0.9500
N1—C1	1.233 (14)	C5—C6	1.447 (15)
N1—C5	1.343 (12)		
			
O1^i^—Co1—O1	90.6 (3)	N1—C1—C2	122.4 (10)
O1^i^—Co1—O1^ii^	90.6 (3)	N1—C1—H1	118.8
O1—Co1—O1^ii^	90.6 (3)	C2—C1—H1	118.8
O1^i^—Co1—N1^i^	85.4 (3)	C3—C2—C1	119.3 (10)
O1—Co1—N1^i^	92.1 (2)	C3—C2—H2	120.4
O1^ii^—Co1—N1^i^	175.1 (3)	C1—C2—H2	120.4
O1^i^—Co1—N1^ii^	92.1 (2)	C4—C3—C2	117.6 (10)
O1—Co1—N1^ii^	175.1 (3)	C4—C3—H3	121.2
O1^ii^—Co1—N1^ii^	85.4 (3)	C2—C3—H3	121.2
N1^i^—Co1—N1^ii^	92.1 (3)	C3—C4—C5	122.2 (11)
O1^i^—Co1—N1	175.1 (3)	C3—C4—H4	118.9
O1—Co1—N1	85.4 (3)	C5—C4—H4	118.9
O1^ii^—Co1—N1	92.1 (2)	N1—C5—C4	121.4 (11)
N1^i^—Co1—N1	92.1 (3)	N1—C5—C6	115.8 (10)
N1^ii^—Co1—N1	92.1 (3)	C4—C5—C6	122.5 (11)
C6—O1—Co1	113.3 (7)	O2—C6—O1	116.2 (13)
C1—N1—C5	117.1 (9)	O2—C6—C5	130.0 (13)
C1—N1—Co1	131.3 (7)	O1—C6—C5	113.8 (10)
C5—N1—Co1	111.5 (7)		
			
O1^i^—Co1—O1—C6	179.0 (6)	C1—C2—C3—C4	−0.2 (14)
O1^ii^—Co1—O1—C6	88.4 (8)	C2—C3—C4—C5	1.2 (15)
N1—Co1—O1—C6	−3.7 (7)	C1—N1—C5—C4	1.3 (13)
O1—Co1—N1—C1	−175.4 (8)	Co1—N1—C5—C4	−177.9 (7)
O1^ii^—Co1—N1—C1	94.2 (9)	C1—N1—C5—C6	176.1 (7)
N1^i^—Co1—N1—C1	−83.4 (7)	Co1—N1—C5—C6	−3.1 (8)
N1^ii^—Co1—N1—C1	8.8 (8)	C3—C4—C5—N1	−1.8 (14)
O1—Co1—N1—C5	3.7 (6)	C3—C4—C5—C6	−176.3 (8)
O1^ii^—Co1—N1—C5	−86.7 (6)	Co1—O1—C6—O2	−177.8 (7)
N1^i^—Co1—N1—C5	95.7 (7)	Co1—O1—C6—C5	2.9 (9)
N1^ii^—Co1—N1—C5	−172.1 (6)	N1—C5—C6—O2	−179.0 (10)
C5—N1—C1—C2	−0.3 (13)	C4—C5—C6—O2	−4.2 (13)
Co1—N1—C1—C2	178.7 (6)	N1—C5—C6—O1	0.2 (9)
N1—C1—C2—C3	−0.2 (14)	C4—C5—C6—O1	174.9 (9)

Symmetry codes: (i) −*y*+1, *x*−*y*+1, *z*; (ii) −*x*+*y*, −*x*+1, *z*.

### Hydrogen-bond geometry (Å, °)

**Table:**

*D*—H···*A*	*D*—H	H···*A*	*D*···*A*	*D*—H···*A*
C3—H3···O2^iii^	0.95	2.60	3.212 (14)	123

Symmetry codes: (iii) *x*−*y*+1, *x*, *z*−1.
